# The Effect of Observational Learning on Self-Efficacy by Sport Competition Condition, Performance Level of Team Members, and Whether You Win or Lose

**DOI:** 10.3390/ijerph191610148

**Published:** 2022-08-16

**Authors:** Taegyong Kwon, Seakhwan Shin, Myoungjin Shin

**Affiliations:** 1 Department of Physical Education, Konkuk University, Seoul 143701, Korea; 2 Department of Leisure Sports, Kangwon National University, Samcheok 25913, Korea

**Keywords:** social cognitive theory, social comparison theory, athletes, attribution

## Abstract

This study examined the effect of athletes’ competition conditions, personal performance level, and attributions toward winning or losing on the relationship between observational learning (OL) and self-efficacy (SE) based on social cognitive theory and social comparison theory. Study 1 verified the validity and reliability of the Korean versions of the Functions of Observational Learning Questionnaire (FOLQ) and the SE Questionnaire. Study 2 investigated differences in the degree to which OL predicts SE in different pressure conditions and personal performance levels. The results showed that OL increased SE in high-performing athletes in high-pressure games and better predicted SE in low-performing athletes in low-pressure games. Study 3 tested the double-mediating effects of effect and OL on the effect of attributions about winning or losing on SE. The results showed that a stronger perception that the cause of winning was internal was associated with increased pride, OL, and SE.

## 1. Introduction

Why do two athletes with similar skills who are taught by the same instructor acquire different techniques? Bandura’s [1] social cognitive theory explains that various human behaviors, learning processes, and learning level differ by the individual’s self-efficacy (SE) level. In social cognitive theory, diverse human behaviors are said to be induced by reciprocal causation among personal determinants, environmental determinants, and behavioral determinants [2,3]. In this study, we tested the effects of SE on learning processes under actual sports conditions, including attributions regarding winning or losing, based on social cognitive theory principles.

### 1.1. The Relationship between SE and OL

SE is the belief in one’s personal ability to accomplish a given achievement [1,4]. To enact a behavior, one must believe that he or she can perform the behavior. Without this belief, the possibility of enacting this behavior decreases. Thus, in social cognitive theory, SE plays an important role in predicting human behavior [4].

An increase in SE is not only a result of experiencing successful performance; it is also influenced by vicarious experiences [4]. Individuals convince themselves that they can successfully perform it if another person can [5,6]. Therefore, OL is another effective instrument for promoting SE [7]. Given this, observing a person similar to oneself successfully performing a task can increase the efficacy of the observer who can master the performance.

Motor learning research has shown that OL induces the acquisition of motor skills and boosts performance levels [8,9]. OL was also found to help develop error detection and correction skills and to strengthen the diversity of motions [10,11,12]. Weiss, McCullagh, Smith, and Berlant [13] reported that OL increased swimming skill performance levels in children with a fear of water by increasing their confidence. Adults who observed the models also experienced improved swimming skills through increased confidence [14]. Therefore, OL can be used as both a cognitive intervention method to improve motor skill acquisition and performance and a useful instrument to promote confidence by emphasizing the motivational aspect.

Applied sports psychology research found that OL has a positive effect on improving SE based on social cognitive theory [15]. According to Bandura [1], SE improves as a result of the following four factors: mastery experience, vicarious experience, social persuasion, and affective and physiological states. Mastery and vicarious experience have the largest impact, suggesting that OL is a combination of mastery experience and vicarious experience. As the similarity between the self and the model increases, SE is strengthened by success and decreased by failure [15]. Empirical research demonstrated that skill acquisition showed greater improvement [16,17], and SE increased more [18,19] when two individuals practiced together (i.e., OL is possible) than when individuals practiced alone (i.e., OL is impossible). 

### 1.2. Motivational Process and Attribution

If there is no motivation to enact the model’s behavior, the behavior is latent and not shown outwardly [20]. The behavior would be enacted when a good result from this behavior is predicted. Furthermore, Bandura [1] (pp. 122–123) stated that attributions regarding success or failure affect the motivational process. Attribution theory categorizes the various causes attributed to winning or losing in the dimensions of casual locus, causal stability, and controllability [21,22], where casual locus induces pride, causal stability induces expectancy, and controllability induces shame.

Combining the above various effects induced from behavioral results can affect the OL motivational processes. For example, an athlete who attributes winning a game to internal, stable, and controllable causes (e.g., one’s ability or effort) experiences increased pride and expectancy and decreased shame, promoting the OL motivational process. Furthermore, Silver, Mitchell, and Gist [23] stated that there is reciprocal causation between attribution and SE and that the cause of the behavioral results can increase or decrease SE. Therefore, the attribution possibly affects the OL motivational process, leading to an increase in SE.

### 1.3. The Relationship between Social Comparison and SE in Sports

According to social comparison theory [24], people tend to compare their own opinions and abilities with others who are similar to them in a self-evaluation. They do this in the absence of criteria to objectively evaluate their opinions and abilities. Social comparison can be divided into three types according to the difference in ability between oneself and the subject, such as upward comparison, which is a comparison with others in a better position than oneself, and similar comparison, which is a comparison with others in a similar position to oneself, and downward comparison, which is a comparison with others in a lesser position than oneself [25,26].

The results of the study in sports and general academic fields showed that the upward comparison had a positive effect on performance [27,28,29]. In the upward comparison, the comparative partner became a model rather than a competitor, which caused the self-improvement motivation, which could have a positive effect on performance [30], and there was a positive relationship with SE [31]. Vrugt and Koenis [31] argued that the upward comparison is based on the comparison target as a model, which increases the self-improvement motivation and increases SE. Carmona, Buunk, Dijkstra, and Peiró [32] also found that upward comparison had a positive effect on SE improvement.

Social comparison is inevitably generated in organizations with similar purposes and abilities [33]: competition-inducing environments and performance-oriented environments promote social comparison [34], and social comparison is mainly generated in environmental contexts with strong uncertainty [35]. In the sports environment, the athletes compete infinitely to achieve the common goal of winning, and there is high uncertainty concerning who will win and lose; thus, social comparison is likely to occur. Therefore, it is desirable to apply social comparison theory to understand the relationship between observational learning and SE in a sports context.

### 1.4. Limitations of Previous Research and Purpose of This Research

Previous studies conducted on OL and SE in the sports field did not reflect the diverse sports environments athletes experience or individual differences in performance levels because they were conducted in laboratory settings. Law and Hall [15] identified problems with previous results of laboratory research and examined the effect of OL on SE by considering the sports environment. Thus, their study was limited in that it did not reflect the elite athlete’s sports environment. Social comparison motivation is strongly induced when the best performance is demanded in competition, and the uncertainty of the situation is high [34,35]. In the sports situation, there are matches with a high competition-related anxiety level and cases where there are not, so the social comparison motivations of athletes may differ, and this merits clarification.

In social cognitive theory, human behavior is produced by reciprocal causation among the personal, environmental, and behavioral determinants. Athletes are exposed to competition (environmental determinant) not only in in-game conditions but also in daily life (e.g., pressure to win). Additionally, because they have different skills, they are categorized into key players and non-key players (personal determinants) within the team. Furthermore, because winning and losing are determined after a game, the impact of OL on SE may differ depending on what the athlete thinks about winning or losing. Based on the social comparison theory, non-key players can have a positive effect on SE by performing an upward comparison with key players as a model, and this relevance is expected to vary depending on the competitive situation.

**Figure 1:**
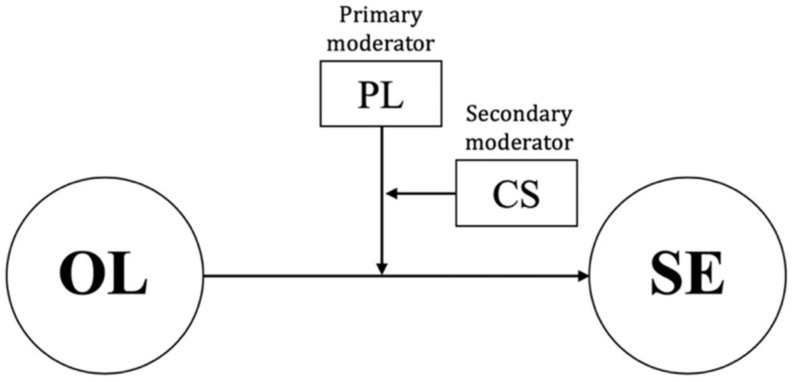
A moderated moderating model. OL, observational learning; SE, self-efficacy; PL, performance level; CS, competition condition.

**Figure 2:**
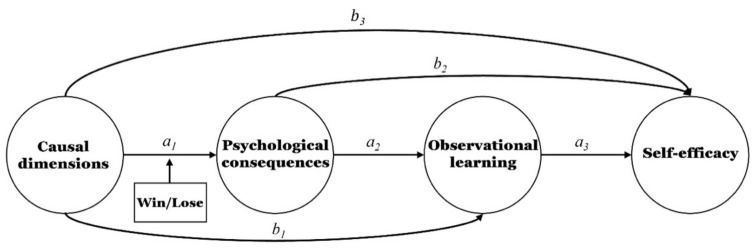
Two serial multiple mediator models.

In the current study, we examined the effects of high-pressure conditions, performance levels, and attribution on the relationship between OL and SE. Study 1 tested the validity of the translated instruments; study 2 investigated the differences in the degree to which OL predicts SE by pressure condition and personal performance level, as shown in Figure 1; study 3 tested the double-mediating effects of effect and OL on the effect of attributions regarding winning or losing on SE, as shown in Figure 2.

## 2. Study 1

### 2.1. Overview

Study 1 measured OL with the functions of the observational learning questionnaire (FOLQ) developed by Cumming, Clark, Ste-Marie, McCullagh, and Hall [36] (2005) and measured SE with the SE questionnaire used by Law and Hall [15]. Because the FOLQ and SE questionnaires were translated for use, study 1 tested the validity and reliability of the translated instruments.

### 2.2. Research Methods

#### 2.2.1. Research Participants

Questionnaires were administered to 211 (199 men) university athletes in (blinded for review) who volunteered to participate and gave written informed consent. The sample included athletes from eight sports (basketball, ice hockey, baseball, archery, tennis, judo, soccer, and rugby), with a mean age of 20.19 years (*SD* = 1.24) and a mean length of sports experience of 9.60 years (*SD* = 2.23). The Research Ethics Committee of each participating university reviewed and approved this study.

#### 2.2.2. Instruments

The FOLQ measures three factors (skill, strategy, and performance) with 17 items on a 7-point scale. The participant reads a description of OL in a sports condition and responds to each measurement item. Based on Cumming and colleagues’ [36] descriptions, we restructured the items to fit the sports environment in Korea as follows: “OL refers to (1) observing another athlete perform a movement or observing the behavior of an athlete in a video; (2) receiving help to increase skill acquisition, performance level, and game management skill while watching a teammate perform; (3) receiving help to increase skill acquisition, performance level, and game management skill while watching a video of another athlete; and (4) receiving help to increase skill acquisition, performance level, and game management skill while watching a video of one’s own play”.

The SE questionnaire measures three factors identical to those of the FOLQ (skill, strategy, and performance), with three items for each factor (nine items total). The participant responds to each item on an 11-point scale of certainty ranging from minimum (0%) to maximum (100%).

The Korean version of the state sport-confidence inventory (SSCI) by Vealey [37] (1986) was used to measure sports confidence. The SSCI measures a single factor with 13 items on a 9-point scale. Confirmatory factor analysis showed that the standardized factor loading for all items was 0.40 or higher and was statistically significant (*p* < 0.001) with 65 degrees of freedom and *χ^2^* = 203.01. Goodness-of-fit was acceptable (TLI = 0.934, CFI = 0.945, RMSEA = 0.080), and Cronbach’s α was 0.96.

The following two items were used to measure participants’ perceived performance level: “I think that the overall percentage of wins in all the games up to now is approximately ___%”, and “I think that the percentage of wins in the games that were important enough to affect my athletic career is approximately ___%”, where the maximum was 100%. The Cronbach’s α was 0.778.

### 2.3. Research Procedure and Analysis Methods

A sports psychologist fluent in English translated the FOLQ and SE questionnaire into Korean, and a translator back-translated them into English. For items that were different from the original document, translation and re-translation were repeated until there were no substantial differences from the original document in the finalized items. Construct validity was examined through confirmatory factor analysis, and convergent validity was tested through the relationships among FOLQ, SE, and SSCI. Reliability was tested through Cronbach’s α. For confirmatory factor analysis, IBM SPSS Statistics, version 18.0 was used to conduct the descriptive analyses. Convergent validity was tested through correlational analysis. The significance level was set at 0.05.

### 2.4. Research Results

#### 2.4.1. Construct Validity and Reliability Testing

To examine the construct validity of the translated FOLQ, first-order confirmatory factor analysis was performed with maximum likelihood. Strategy item 5, which showed a factor load of 0.40 or below, was eliminated, and a second-order confirmatory factor analysis was performed. The results showed statistical significance with 101 degrees of freedom and *χ^2^* = 311.66 (*p* < 0.001), with acceptable goodness-of-fit (TLI = 0.924, CFI = 0.909, RMSEA = 0.083). Thus, construct validity was confirmed. The internal consistency (α) was 0.94 for skill, 0.89 for strategy, and 0.91 for performance. The confirmatory factor analysis on the translated SE questionnaire showed statistical significance with 24 degrees of freedom and *χ^2^* = 96.07 (*p* < 0.001) and acceptable goodness-of-fit (TLI = 0.937, CFI = 0.958, RMSEA = 0.080). Thus, construct validity was confirmed. The internal consistency (α) was 0.88 for skill, 0.90 for strategy, and 0.91 for performance.

#### 2.4.2. Convergent Validity and Predictive Validity

SSCI is the belief in the success of one’s performance in a sports condition and is similar to SE [37]. Thus, a relationship with both SE and OL should exist (convergent validity testing). Furthermore, because SE is a major variable for predicting behavior in social cognitive theory, the personal performance level should also have a relationship with SE (predictive validity testing).

As shown in Table 1, SSCI was significantly correlated with three SE factors (skills, strategies, and performance level) and three OL factors (skills, strategies, and performance level), and the SE factor correlations tended to be higher than the OL factor correlations. The perceived performance level only had statistically significant correlations for three SE factors.

## 3. Study 2

### 3.1. Overview

Study 2 examined the conditional effect of the interaction between personal performance level and OL on SE by competition condition. The specific research hypotheses were:

**Hypothesis** **1** **(H1).** *For a key player in the team, the effect of OL on SE will strengthen in a high-pressure condition*.

**Hypothesis** **2** **(H2).** *For a non-key player in the team, the effect of OL on SE will weaken in a high-pressure condition*.

### 3.2. Research Methods

#### 3.2.1. Research Participants

Questionnaires were given to 136 university athletes (131 men, 5 women) across seven sports (soccer, baseball, basketball, tennis, archery, taekwondo, and table tennis) who volunteered to participate and gave written informed consent. The mean participant age was 20.24 years (*SD* = 1.33), and the mean length of sports experience was 9.78 years (*SD* = 2.20). The Research Ethics Committee of each participating university reviewed and approved this study.

#### 3.2.2. Instruments

The FOLQ and SE questionnaire were used, for which the validity and reliability were confirmed in study 1. The mean values of the three FOLQ and SE questionnaires’ factors were used in the final analysis.

#### 3.2.3. Proportion of Being in the Game

Each participant’s proportion of being in the game was measured with the following two items: (1) What is the proportion of games in which you played on the field rather than being on the bench with your current team? (%); (2) what is the proportion of important games (e.g., finals, games where winning was essential) in which you played for your current team? (%). Participants who played solo sports responded to the items assuming team games. Athletes with higher proportions of being in the game were assumed to have a higher team contribution and performance level than athletes with lower proportions of being in the game. The Cronbach’s α was 0.89.

#### 3.2.4. Covariates

Perception of pressure, which was the manipulation task in this study, can differ by the trait anxiety level of each participant. Therefore, competitive trait anxiety was measured and used as a control variable. To measure each participant’s competitive trait anxiety, we used the sports competition anxiety test (SCAT), developed by Martens, Vealey, and Burton [38]. The SCAT includes 15 items on a 3-point scale (1: usually not true; 2: sometimes true; 3: often true). Five items are not calculated in the score. The Cronbach’s α for the other ten items was 0.75. To identify whether participants’ basic characteristics were randomly assigned to high- and low-pressure conditions, age and sports experience (years) were included in the analyses.

#### 3.2.5. Research Process and Analysis Methods

Competitions were categorized into high-pressure and low-pressure conditions. For high-pressure conditions, each participant was asked which game in the past two years had the highest pressure or tension and why, and SE was measured for the high-pressure games. For low-pressure conditions, each participant was asked which game in the past two years had the lowest pressure, was unimportant, and why, and the SE for that game was used.

Participants were randomly assigned to the high-pressure group (*n* = 69) or the low-pressure group (*n* = 67). Then, the effect of usual OL on SE that changes with sports competition conditions was examined. The order of the items measured in the inventory were basic characteristics, the proportion of being in the game, competitive trait anxiety, and OL followed by the SE measurement by competition condition manipulation. 

The sports competition conditions were dummy coded. IBM SPSS Statistics, version 18.0 was used to conduct all analyses. A MANOVA was conducted to test homogeneity between the high- and low-pressure groups. PROCESS [39] was used to examine the moderated mediation effect of the competition condition (secondary moderator) on the conditional interaction effect between OL and personal performance level on SE. To calculate a standardized regression coefficient, the entered variables were standardized before performing the analysis. The statistical significance level was set at 0.05.

### 3.3. Results

#### 3.3.1. Basic Statistical Analysis

Descriptive statistics and correlations are shown in Table 2. Since the correlation among the entered variables was not high, multicollinearity was considered low. Skewness and kurtosis were within ±2. A Q-Q plot showed that most of the observed data were linear. Thus, the variables were considered normally distributed.

#### 3.3.2. Homogeneity Test

To test the random assignment between the high-pressure group (*n* = 69) and the low-pressure group (*n* = 67), between-group differences in age, length of sports experience, the proportion of being in the game, and trait anxiety were examined through the MANOVA. The results showed that Wilks’ lambda was not statistically significant at 0.02, *F* (4, 131) = 0.67 (*p* = 0.608). Therefore, there was no difference between the two groups in terms of age, length of sports experience, the proportion of being in the game, and trait anxiety. Thus, the participants who were categorized by a winning or losing condition did not significantly differ on basic characteristics, the proportion of being in the game, or trait anxiety.

#### 3.3.3. Moderated Mediation Effect

The moderated mediation effect of the competition condition (secondary moderator) on the conditional effect of the interaction between OL and the proportion of being in the game (primary moderator) on SE was tested. The results showed that 34% (R^2^ = 0.34) of the total distribution of the SE dependent variable was explained by the variables entered in the model (CV, IV, PMV, and SMV) as shown in Table 3, and this was statistically significant (*F* (12, 123) = 5.34, *p* < 0.001). The slopes of the three-way interaction variables (*t* = 2.08, *p* < 0.05) ∆R^2^ (*F* (1, 123) = 4.34, *p* < 0.05) were all statistically significant.

Post hoc simple regression analysis was performed to interpret the three-way interaction effect. First, examining the impact of the interaction between the proportion of being in the game and OL on SE showed that OL negatively affected SE (β = −0.49) in participants with a low proportion of being in the game (the mean proportion of being in the game minus 1 *SD*) but that OL had a positive effect on SE (β = 0.40) in participants with a high proportion of being in the game (the mean proportion of being in the game plus 1 *SD*). However, the conditional effect of the interaction between the proportion of being in the game and OL on SE differed by competition condition. In the low-pressure group, the slope (β) of the effect of OL on SE in participants with a low proportion of being in the game was statistically significant at 0.79 (*t* = 3.46, *p* < 0.001). In contrast, the slope (β) of the participants with a high proportion of being in the game was 0.12 (*t* = 0.79, *p* = 0.434) and not statistically significant. In the high-pressure group, the slope (β) of the effect of OL on SE in participants with a low proportion of being in the game was 0.30 (*t* = 1.74, *p* = 0.084) and not statistically significant, but the slope (β) of the participants with a high proportion of being in the game was 0.52 (*t* = 3.07, *p* < 0.01) and statistically significant. Therefore, OL predicted SE more strongly in participants with a high proportion of being in the game in a high-pressure competition, while OL predicted SE more strongly in participants with a low proportion of being in the game in a low-pressure competition.

## 4. Study 3

### 4.1. Overview

Because wins and losses are relatively clear in a sports environment, the OL motivational process can be affected by athletes’ attributions regarding a game’s outcome, leading to a different effect on SE. Most sports-related attribution research has focused on improving performance levels by resetting the attribution [40,41,42] and thus does not examine how athletes’ attributions lead to performance level improvements. In study 3, the effect of OL on SE by attribution was tested to show the psychological process by which attribution affects performance level improvement. The specific hypotheses were:

First, the effect of causal dimensions on effect will differ by winning or losing a game (Figure 2). Second, there are double-mediating effects of effect and OL on the attribution effect for winning or losing on SE (Figure 2). 

### 4.2. Research Methods

#### 4.2.1. Research Participants

In this study, questionnaires were administered to 165 university athletes (132 men), who volunteered to participate and gave written informed consent, across 11 sports (wrestling, badminton, bowling, boxing, shooting, cycling, swimming, archery, taekwondo, tennis, and squash). The mean age of the participants was 20.31 years (*SD* = 1.33), and the mean length of sports experience was 9.56 years (*SD* = 2.55). The Research Ethics Committee of each participating university reviewed and approved this study.

#### 4.2.2. Instruments

The FOLQ and SE questionnaire were used as described in study 2. To measure attributions regarding the game outcome (winning or losing), we revised the Causal Dimension Scale for Teams (CDS-T) by Greenlees and colleagues [42] to fit the purpose of this study. Although the CDS-T was developed to measure team attribution, most items were made regarding the items in the Revised Causal Dimension Scale (CDS II) developed by McAuley, Duncan, and Russell [43], which measures four factors (locus of causality, stability, team controllability, and external control) with 16 items on a 9-point scale. Higher scores indicate higher internal locus, stability, and external control. Because the CDSII is a general personal attribution measurement rather than specific to sports, CDS-T was deemed more appropriate for the current study. The word “team” was changed to “individual” or “self” in the items. The questionnaire was administered using 12 items across three factors after excluding four items on team controllability.

Participants were randomly assigned to one of two groups, defined as winning or losing, and asked to identify which competition in the past two years had the highest pressure or tension and why. Group A (winning) recorded four causes for winning, and group B (losing) recorded four causes for losing; then, both groups completed the CDS-T. 

First-order confirmatory factor analysis results were statistically significant, with 51 degrees of freedom and *χ^2^* = 145.447 (*p* < 0.001); however, the goodness-of-fit was not optimal (TLI = 0.804, CFI = 0.848, RMSEA = 0.106). One item on locus of causality, one item on stability, and one item on external control that each showed factor loading of 0.40 or below were eliminated, and a second-order confirmatory factor analysis was performed. The results were statistically significant, with 24 degrees of freedom and *χ^2^* = 46.74 (*p* < 0.001), and goodness-of-fit met the acceptable threshold (TLI = 0.926, CFI = 0.951, RMSEA = 0.076). Cronbach’s α was 0.84 for locus of causality, 0.75 for stability, and 0.72 for external control.

#### 4.2.3. Psychological Results

Based on Weiner’s [21] attribution theory, three psychological consequences of pride, shame, and expectancy were measured through eight items. Pride was measured with the following four items on a 5-point scale: (1) (After the game ended) I felt that I was at least as valuable as others; (2) (after the game ended) I felt that I had a lot of strengths (qualifications); (3) (after the game ended) I was generally satisfied with myself; (4) (after the game ended,) I felt that I was competent. Shame was measured with the following three items on a 5-point scale: (1) (After the game ended) I was ashamed to see people around me, such as my parents, instructor, or teammates; (2) (after the game ended) I felt shame; (3) (after the game ended) I felt that people around me, such as my parents, instructor, or teammates, saw me as being pathetic. Expectancy was measured with the following item on a 100-point scale: (1) If you met your opponent again (after the game), how likely is it that you would win? ___%, with a maximum of 100%. The Cronbach’s α was 0.88 for pride and 0.88 for shame.

#### 4.2.4. Proportion of Being in the Game

Each participant’s proportion of being in the game was measured with the following two items: (1) What is the proportion of games in which you played on the field rather than being on the bench in your current team? (%); (2) what is the proportion of important games (e.g., finals, games where winning was essential) in which you played in your current team? (%). Participants who played solo sports (e.g., archery, judo, taekwondo, and tennis) responded to the items assuming team games. Athletes with a higher proportion of being in the game were assumed to have a higher contribution to the team and higher performance level than athletes with lower proportions of being in the game. The Cronbach’s α was 0.89.

#### 4.2.5. Covariates

The proportion of being in the game, age, and length of sports experience (years) were measured using the same items as study 2. The Cronbach’s α for the proportion of being in the game was 0.82.

#### 4.2.6. Research Procedure and Analysis Methods

For athletes who consented to participate in this study, basic background information, the proportion of being in the game, and OL were measured. Next, winning and losing conditions in the competition with the largest tension in the past two years (structured based on the results of study 2) were randomly presented. Then, the attribution to the winning/losing condition, psychological consequences, and SE were each measured. 

IBM SPSS Statistics, version 18.0 was used to conduct all analyses. A MANOVA was used to test the homogeneity between the winning and losing conditions. Two serial multiple mediator models by the winning and losing conditions are presented in Figure 2. Model 83 in the PROCESS [39] was used. To calculate the standardized regression coefficients, the entered variables were standardized and analyzed. The statistical significance level was set at 0.05.

## 5. Results

### 5.1. Basic Statistical Analysis

Descriptive statistics and correlational analysis are shown in Table 4. Since the correlation among the entered variables was not high, multicollinearity was considered low. Skewness and kurtosis were within ±2. The Q-Q plot also showed that most of the observed data were linear. Thus, the variables were considered normally distributed. 

### 5.2. Homogeneity Test

To test for random assignment between the winning condition group (*n* = 87) and the losing condition group (*n* = 78), between-group differences in age, length of sports experience, and proportion of being in the game was examined through a MANOVA model. The results showed that Wilks’ lambda was not statistically significant at 0.965, *F* (3, 161) = 1.93 (*p* = 0.127). Therefore, there were no between-group differences in age, length of sports experience, or proportion of being in the game. Thus, there were no significant differences in basic characteristics or proportion of being in the game between the winning and losing groups

#### 5.2.1. The Effect of Causal Locus on SE Mediated by Pride and OL in Winning and Losing Conditions

After entering sex, age, length of sport experience, and proportion of being in the game as covariates (Table 5), differences in the double-mediating effects of pride and OL on the effect of casual locus on SE in winning and losing conditions were tested as shown in Figure 2. The results showed that the effect (a_1_) of casual locus on pride (MV1) was 0.43 (*t* = 4.12, *p* < 0.001), and R^2^ was 0.35 (*F* (7, 157) = 12.32, *p* < 0.001), which was statistically significant. The effect (b_1_) of casual locus on OL (MV2) was 0.17 (*t* = 2.29, *p* < 0.05), and the effect (a_2_) of pride on OL was 0.41 (*t* = 5.36, *p* < 0.001), and R^2^ was 0.20 (*F* (6, 158) = 6.69, *p* < 0.001), which was statistically significant. Finally, the standardized regression coefficients of the effect of casual locus (b_3_), pride (b_2_), and OL (a_3_) on SE (DV) were 0.03 (*t* = 0.51, *p* = 0.606), 0.35 (*t* = 5.38, *p* < 0.001), and 0.46 (*t* = 7.38, *p* < 0.001), respectively. R^2^ was 0.52, which was statistically significant (*F* (7, 157) = 24.31, *p* < 0.001). 

Furthermore, the slope of IT (a × b) was −0.74 (*t* = 5.61, *p* < 0.001), and ΔR^2^ was 0.13 (*F* (1, 157) = 31.52, *p* < 0.001), which was statistically significant. Thus, the effect of a casual locus on pride differed between the winning and losing conditions. To examine differences in the effect of a casual locus on pride between the winning and losing conditions, simple slopes were analyzed to probe for an interaction. The effect of casual locus on pride in a winning condition was 0.43 (*t* = 4.11, *p* < 0.001), while the effect in a losing condition was −0.31 (*t* = 3.69, *p* < 0.001), showing effects in opposite directions. In other words, more internal attribution to winning was associated with increased pride, while more internal attribution to losing was associated with decreased pride. 

Using the bootstrap method, we tested for differences in the indirect effect of the winning or losing condition on SE (casual locus → pride (MV1) → OL (MV2) → SE). The results showed that the indirect effect of the two serial multiple mediators was 0.08 in a winning condition and –0.06 in a losing condition. Because neither result included “0” in the 95% confidence interval, both winning and losing conditions had a statistically significant indirect effect on the two serial multiple mediators. Therefore, higher internal attributions to winning were associated with increased SE mediated by pride and OL, while more internal attributions to losing were associated with decreased SE mediated by pride and OL. 

#### 5.2.2. The Effect of External Control on SE Is Mediated by Shame and OL in Winning and Losing Conditions

After entering sex, age, length of sport experience, and proportion of being in the game as covariates (Table 6), differences in the double-mediating effects of shame and OL on the effect of external control on SE in winning and losing conditions were tested, as shown in Figure 2. The results showed that the effect (a_1_) of external control on shame (MV1) was −0.10 (*t* = 1.01, *p* = 0.314) and not statistically significant but that R^2^ was 0.15 (*F* (7, 157) = 4.11, *p* < 0.001) and statistically significant. The effect (b_1_) of external control on OL (MV2) was 0.00 (*t* = *0*.05, *p* = 0.958), and the effect (a_2_) of shame on OL was −0.13 (*t* = 1.65, *p* = 0.101), and R^2^ was 0.05 (*F* (6, 158) = 1.37, *p* = 0.229), so none were statistically significant. The standardized regression coefficients for the effect of external control (b_3_), shame (b_2_), and OL (a_3_) on SE (DV) were −0.06 (*t* = 0.98, *p* = 0.324), −0.22 (*t* = 3.64, *p* < 0.001), and 0.56 (*t* = 9.48, *p* < 0.001), respectively. R^2^ was 0.48, which was statistically significant (*F* (7, 157) = 20.66, *p* < 0.001). 

Furthermore, the slope of IT (c × d) was 0.36 (*t* = 2.41, *p* < 0.05), and ΔR^2^ was 0.03 (*F* (1, 157) = 5.82, *p* < 0.05), which was statistically significant. Thus, the effect of external control on shame differed between winning and losing conditions. To examine this difference, simple slopes were analyzed to probe for an interaction. The results showed that the effect of external control on shame in a winning condition was –0.10 (*t* = 1.01, *p* = 0.314), while the effect in a losing condition was 0.26 (*t* = 2.35, *p* < 0.05), so they moved in opposite directions. In other words, more external and controllable attribution to winning was associated with decreased shame, while more external and controllable attribution to losing was associated with increased shame.

Using the bootstrap method, we tested differences in indirect effects of the winning or losing condition on SE (external control→ shame (MV1) → OL (MV2) → SE). The results showed that the indirect effect of the two serial multiple mediators was 0.01 in a winning condition and −0.02 in a losing condition. Because both include “0” in the 95% confidence interval, neither the winning nor losing condition had a statistically significant indirect effect.

## 6. Discussion

### 6.1. Validity and Reliability of the Instrument

As a result of study 1’s validity and reliability testing on the translated FOLQ and SE questionnaires, one item was eliminated from FOLQ (“I use OL to develop game plans and routines.”) It is possible that factor loading did not meet the threshold because the term “routine” was not familiar to Korean athletes. 

In Table 1, SSCI was correlated with three SE factors and three OL factors; however, correlations with the three SE factors were higher than correlations with the three OL factors. Since Vealey [37] defined sports confidence as the belief in the personal performance level formed in competition conditions, it is natural that the correlation between SSCI and SE is higher than between SSCI and OL. Furthermore, because many studies have found that SE is a major predictor of performance level [44,45], the existence of a relationship between the three SE factors and the perceived performance level supports the results of previous research. 

### 6.2. Effect of OL on SE

In study 2, the effects of OL on SE by competition condition and participants’ performance level were tested based on social cognitive theory and social comparison theory. As shown in Table 2, there was a correlation between OL and SE. Moreover, as shown in Table 3, OL was found to predict SE. In line with the results of this study, Law and Hall [15] also observed a positive effect of OL on SE. Furthermore, given that SE can be strengthened by mastery and vicarious experiences and OL is a form of mastery and vicarious experience [1], the results of this study support the social cognitive theory. 

Athletes who are key players and have a high proportion of playing time in important games are likely to be high-performing athletes. In study 2, the proportion of playing time in games was used as the standard to distinguish between high-performing and lower-performing athletes. Results showed that OL helped improve SE in high-performing athletes in high-pressure games but did not affect SE improvement in lower-performing athletes. In contrast, OL was a stronger predictor of SE in low-performing than high-performing athletes in low-pressure games. 

In summary, positive psychological phenomena were observed in key players in high-pressure games and non-key players in low-pressure games. The cause of such results seems to be a phenomenon caused by different social comparison types; e.g., in low-pressure games, the non-key player is upward assimilative, and in high-pressure games, the key player is downward contrastive. Upward-assimilative experiences foster feelings of optimism and respect for others, and downward-contrastive experiences foster feelings of pride in oneself and pleasure in the unhappiness of subordinates [46]. When this is applied to the results of this study, in low-pressure games, non-key players had respect for the key player and a generous evaluation of their own performance, which led to improved SE by observing a key player. On the other hand, in high-pressure games, a key player can increase their self-esteem in their abilities by comparing them with a non-key player, so it is used as a strategy to improve their SE by observing the mistakes or incorrect performance of lower performers.

#### The Double-Mediating Effects of Psychological Consequences and OL on the Effect of Causal Dimension on SE 

In study 3, we tested the double-mediating effects of effect and OL on the effect of attributions regarding wins and losses on SE in different pressure conditions. The results showed that a stronger perception of an internal cause of winning was associated with increased pride and also, sequentially, OL and SE. However, the indirect effect of psychological consequences on stability and controllability mediated by OL on SE was not statistically significant. Therefore, OL is affected by causal locus in the attribution dimension (i.e., casual locus, stability, and controllability) in the motivational process. OL appears to be strengthened by pride among the three effect types (i.e., pride, expectancy, and shame) induced by the causal dimensions. Information learned through observation is latent and not active until the observer has a reason to use it [2]. Thus, a psychological counseling strategy that can strengthen and maintain pride may help increase OL.

Result 4 in study 3 showed a direct effect of external control on shame by condition (winning or losing) but no significant indirect effect that strengthens SE mediated by OL. However, this result supports Weiner’s [21] final attribution model, where one must attribute the cause of winning to internal and controllable factors (e.g., effort) rather than internal and uncontrollable factors (e.g., natural talent) and, conversely, the cause of losing to external and uncontrollable factors (e.g., luck) rather than external and controllable factors (e.g., bias and severity of the instructor or the referee). (Because the bias or the strictness of a teacher or a referee can be controlled by them, they are seen as external but controllable aspects [20].) One interpretation of the interaction effect shown in Table 6 is that more external and controllable attributions to losing were associated with increased shame, which supports Weiner’s [21] results.

### 6.3. Limitations and Future Directions

Although significant results were found, there are several limitations. First, most participants in this study were male athletes, and the research could not be conducted on athletes in all sports, so the results of the study may not be generalizable. Follow-up studies are needed that include more female athletes and diverse sports to ensure the generalizability of future results. 

George, Deborah, Feltz, and Chase [47] tested the effect of OL on SE depending on whether the model in the video presented to novice exercisers was a novice (i.e., high model similarity) or an expert (i.e., low model similarity). The results showed that high model similarity positively affected SE and exercise performance compared to low model similarity. Given that the participant pool used in George and colleagues’ [47] study comprised the general population rather than athletes, future studies should test the sequential effect that model similarity has on OL, SE, and exercise performance in athletes.

Weiner [21] categorized the controllability dimension as controllable and uncontrollable within the internal factors and controllable and uncontrollable within the external factors. However, because this study used the Causal Dimension Scale for Teams (CDS-T) used by Greenlees and colleagues [42], only the team controllability factor was eliminated, and thus, the personal controllability factor could not be measured. In future studies, it will be necessary to develop an attribution measurement instrument that is suitable for solo sports to further investigate the internal controllability dimension. 

## 7. Conclusions

OL based on humans’ vicarious learning capability is known to strengthen SE [6]. Therefore, the effect of OL on SE based on a personal performance level and pressure condition was evaluated in study 2 based on the social cognitive and social comparison theories. It was found that the relationship between OL and SE varied according to the competition conditions and perceived performance level of team members. In high-pressure games, the positive relationship between OL and SE was strengthened in high performers, whereas the same phenomenon was also observed in low performers in low-pressure games. In addition, Bandura [1] stated that attributions regarding success and failure affected the OL motivational process. Since wins and losses are clear in a sports environment, the two serial multiple mediating effects of the attribution to wins and losses that sequentially affected OL and SE were evaluated in study 3. As a result, in the case of the cause of winning as internal and cause of losing as external, the individual’s pride increased, and OL and SE were also sequentially strengthened. Through this study, the relationship between OL and SE was found to be affected by sports competition environment and individual cognitive/psychological factors. Thus, these results supported the social cognitive theory and social comparison theory.

## Figures and Tables

**Table 1:** Correlation Coefficients and Perceived Performance Level.

	1	2	3	4	5	6	7	8
1. SSCI	1							
2. SE for skills	0.62 ***	1						
3. SE for strategies	0.60 ***	0.84 ***	1					
4. SE for performance level	0.55 ***	0.76 ***	0.72 ***	1				
5. Skill OL	0.32 ***	0.39 ***	0.38 ***	0.36 ***	1			
6. Strategy OL	0.30 ***	0.39 ***	0.38 ***	0.31 ***	0.72 ***	1		
7. Performance level OL	0.25 ***	0.25 ***	0.27 ***	0.24 **	0.38 ***	0.54 ***	1	
8. Perceived performance level	0.51 ***	0.37 ***	0.41 ***	0.34 ***	0.12	0.08	0.03	1

Note: SSCI, state sport-confidence inventory; SE, self-efficacy; OL, observational learning. ** *p* < 0.01, *** *p* < 0.001.

**Table 2:** Descriptive Statistical Analysis.

	1	2	3	4	5	6
1. Age	-					
2. Sport experience	0.42 ***	-				
3. Proportion of being in the game	0.24 **	0.19 *	-			
4. OL	0.03	0.02	0.11	-		
5. SE	0.07	0.21 *	0.36 ***	0.40 ***	-	
6. Competitive trait anxiety	−0.00	−0.06	−0.01	−0.05	−0.01	-
Mean	20.24	9.78	49.53	5.08	72.57	2.01
SD	1.33	2.20	36.18	0.96	15.35	0.46
Skewness	−0.40	−0.28	0.08	0.68	−0.32	−0.00
Kurtosis	1.51	0.09	−1.45	−0.43	0.73	−0.38

Note: SE, self-efficacy; OL, observational learning. * *p* < 0.05, ** *p* < 0.01, *** *p* < 0.001.

**Table 3:** The Moderated Mediation Effect.

	DV	SE	
		β	R^2^ (∆R^2^ for a × b × c)
CV	Sex	0.12	0.34 *** (0.02 *)
	Age	0.03	
	Length of sport experience	0.13	
	Competitive trait anxiety	−0.02	
IV	OL ^a^	0.46 ***	
PMV	Proportion of being in the game ^b^	−0.09	
SMV	Competition condition ^c^	0.35 **	
IT	a × b	−0.26 *	
	a × c	−0.05	
	b × c	−0.19	
	a × b × c	0.36 *	

Note. DV, dependent variable; CV, covariate variable; IV, independent variable; PMV, primary moderator variable; SMV, secondary moderator variable; SE, self-efficacy; OL, observational learning; IT, interaction term; ^a^ OL; ^b^ proportion of being in the game; ^c^ competition condition. * *p* < 0.05, ** *p* < 0.01, *** *p* < 0.001.

**Table 4:** Descriptive Statistical Analysis.

	1	2	3	4	5	6	7	8	9	10	11	12
1. Age	-											
2. Length of sport experience	0.42 ***	-										
3. Proportion of being in the game.	0.03	0.05	-									
4. OL	−0.12	0.03	0.104	-								
5. SE	−0.12	−0.09	0.27 ***	0.61 **	-							
6. Locus of causality	−0.12	−0.06	0.24 ***	0.19 *	0.18 *	-						
7. Stability	−0.01	0.01	0.12	0.06	0.05	0.17 *	-					
8. Team controllability	−0.07	−0.01	0.22 **	0.26 **	0.35 ***	0.52 ***	0.421 ***	-				
9. External control	−0.074	0.08	0.001	0.02	−0.05	0.30 ***	0.11	0.11	-			
10. Pride	−0.22 **	−0.19 *	0.27 ***	0.40 **	0.57 ***	0.09	0.17 *	0.31 ***	0.02	-		
11. Shame	0.02	0.18 *	−0.11	−0.12	−0.32 ***	−0.12	−0.03	−0.20 *	0.05	−0.30 ***	-	
12. Expectancy	−0.11	−0.13	0.28 ***	0.43 **	0.52 ***	0.02	−0.02	0.17 *	−0.16 *	0.44 ***	−0.24 **	-
Mean	20.31	9.56	68.40	4.92	67.42	5.81	4.95	5.37	5.57	3.24	2.56	37.42
SD	1.33	2.55	31.95	0.89	17.24	1.50	1.40	1.21	1.12	0.81	1.01	10.97
Skewness	0.21	−0.00	4.92	−0.11	−0.62	−0.13	0.05	−0.22	0.08	−0.05	0.08	−0.76
Kurtosis	0.41	0.73	0.89	0.41	1.05	−0.06	0.29	−0.06	0.40	−0.02	−0.72	0.43

Note: SE, self-efficacy; OL, observational learning. * *p* < 0.05, ** *p* < 0.01, *** *p* < 0.001.

**Table 5:** Analysis 1: Double-Mediating Effects in Winning and Losing Conditions.

		Pride (MV 1)		OL (MV 2)		SE (DV)	
		Path	β	Path	β	Path	β
COV	Sex		0.28		−0.06		0.04
	Age		−0.16 *		−0.07		0.04
	Length of sport experience		−0.08		0.15		−0.05
	Proportion of being in the game		0.24 ***		−0.05		0.13 *
MOV	Win or loss ^a^		−0.57 ***				
IV	Causal locus ^b^	a_1_	0.43 ***	b_1_	0.17 *	b_3_	0.03
	Pride			a_2_	0.41 ***	b_2_	0.35 ***
	OL					a_3_	0.46 ***
IT	a×b		−0.74 ***				
	R^2^ (ΔR^2^)	0.35 *** (0.13 ***)		0.20 ***		0.52 ***	

Note. Winning and losing conditions (Winning = 0, Losing = 1); MV, mediator variable; DV, dependent variable; IV, independent variable; COV, covariate; MOV, moderator variable; IT, interaction term; SE, self-efficacy; OL, observational learning; ^a^ win or loss; ^b^ causal locus. * *p* < 0.05, *** *p* < 0.001.

**Table 6:** Analysis 2: Double-Mediating Effects in Winning and Losing Conditions.

		Shame (MV 1)		OL (MV 2)		SE (DV)	
		Path	β	Path	β	Path	β
COV	Sex		0.02		0.08		0.14
	Age		−0.11		−0.17 *		−0.04
	Length of sport experience		0.19 *		0.11		−0.06
	Proportion of being in the game		−0.11		0.09		0.20 ***
MOV	Win or loss ^c^		0.54 ***				
IV	External control ^d^	a_1_	−0.10	b_1_	0.00	b_3_	−0.06
	Shame			a_2_	−0.13	b_2_	−0.22 ***
	OL					a_3_	0.56 ***
IT	c×d		−0.36 *				
	R^2^ (ΔR^2^)	0.15 *** (0.03 *)		0.05		0.48 ***	

Note: Winning and losing conditions (Winning = 0, Losing = 1); MV, mediator variable; DV, dependent variable; IV, independent variable; COV, covariate; MOV, moderator variable; IT, interaction term; SE, self-efficacy; OL, observational learning; ^c^ win or loss; ^d^ external control. * *p* < 0.05, *** *p* < 0.001.

## References

[1] Bandura A. (1997). Self-Efficacy: The Exercise of Control.

[2] Bandura A. (1986). Social Foundations of Thought and Action: A Social Cognitive Theory.

[3] Stajkovic A.D., Luthans F., Porter L.W., Bigley G.A., Steers R.M. (2003). Social cognitive theory and self-efficacy: Implications for motivation theory and practice. Motivation and Work Behavior.

[4] Bandura A., Pajares F., Urdan T. (2006). Guide for constructing self-efficacy scales. Self-Efficacy Beliefs of Adolescents.

[5] Bandura A. (1982). The assessment and predictive generality of self-percepts of efficacy. J. Behav. Ther. Exp. Psychiatry.

[6] Schunk D.H., Hanson A.R., Cox P.D. (1987). Peer-model attributes and children’s achievement behaviors. J. Educ. Psychol..

[7] Lee S., Kwon S., Ahn J. (2021). The effect of modeling on self-efficacy and flow state of adolescent athletes through role models. Front. Psychol..

[8] McCullagh P., Weiss M.R., Singer R.N., Hausenblas H.A., Janelle C.M. (2001). Modeling: Considerations for motor skill performance level and psychological responses. The Handbook of Sport Psychology.

[9] Williams A.M., Davids K., Williams J.G., Williams A.M., Davids K., Williams J.G. (1999). Observational learning. Visual Perception and Action in Sport.

[10] Black C.B., Wright D.L. (2000). Can observational practice facilitate error recognition and movement production?. Res. Q. Exerc. Sport.

[11] Blandin Y., Proteau L. (2000). On the cognitive basis of observational learning: Development of mechanisms for the detection and correction of errors. Q. J. Exp. Psychol..

[12] Hodges N.J., Chua R., Franks I.M. (2003). The role of video in facilitating perception and action of a novel coordination movement. J. Motor. Behav..

[13] Weiss M.R., McCullagh P., Smith A.L., Berlant A.R. (1998). Observational learning and the fearful child: Influence of peer models on swimming skill performance level and psychological responses. Res. Q. Exerc. Sport.

[14] Starek J., McCullagh P. (1999). The effect of self-modeling on the performance level of beginning swimmers. Sport Psychol..

[15] Law B., Hall C. (2009). Observational learning use and self-efficacy beliefs in adult sport novices. Psychol. Sport Exerc..

[16] Arripe-Longueville F., Fleurance P., Winnykamen F. (1995). Effects of the degree of competence symmetry-asymmetry in the acquisition of a motor skill in a dyad. J. Hum. Mov. Stud..

[17] Shea C.H., Wulf G., Whitacre C. (1999). Enhancing training efficiency through the use of dyad training. J. Motor Behav..

[18] Legrain P., d’Arripe-Longueville F., Gernigon C. (2003). The influence of trained peer tutoring on tutors’ motivation and performance level in a French boxing setting. J. Sports Sci..

[19] Legrain P., d’Arripe-Longueville F., Gernigon C. (2003). Peer tutoring in a sport setting: Are there any benefits for tutors?. Sport Psychol..

[20] Bandura A. (2001). Social cognitive theory of mass communication. Media Psychol..

[21] Weiner B. (2010). The development of an attribution-based theory of motivation: A history of ideas. Educ. Psychol..

[22] Martinko M.J., Mackey J.D. (2019). Attribution theory: An introduction to the special issue. J. Organ. Behav..

[23] Silver W.S., Mitchell T.R., Gist M.E. (1995). Responses to successful and unsuccessful performance: The moderating effect of self-efficacy on the relationship between performance and attributions. Organ. Behav. Hum. Decis. Process..

[24] Festinger L. (1954). A theory of social comparison processes. Hum. Relat..

[25] Michinov N. (2007). Social comparison and affect: A study among elderly women. J. Soc. Psychol..

[26] Miyake M., Matsuda F. (2002). Effects of generalized self-efficacy and negative social comparison feedback on specific self-efficacy and performance. Psychol. Rep..

[27] Dijkstra P., Kuyper H., Van der Werf G., Buunk A.P., van der Zee Y.G. (2008). Social comparison in the classroom: A review. Rev. Educ. Res..

[28] Diel K., Broeker L., Raab M., Hofmann W. (2021). Motivational and emotional effects of social comparison in sports. Psychol. Sport. Exerc..

[29] Xing H., Yao M., Zhu W., Li J., Liu H. (2022). The role of perceived parent social comparisons in adolescent academic social comparison, self-efficacy, and self-handicapping: A person-centered approach. Curr. Psychol..

[30] Wills T.A. (1981). Downward comparison principles in social psychology. Psychol. Bull..

[31] Vrugt A., Koenis S. (2002). Perceived self-efficacy, personal goals, social comparison, and scientific productivity. Appl. Psychol..

[32] Carmona C., Buunk A.P., Dijkstra A., Peiró J.M. (2008). The relationship between goal orientation, social comparison responses, self-efficacy, and performance. Eur. Psychol..

[33] Greenberg J., Ashton-James C.E., Ashkanasy N.M. (2007). Social comparison processes in organizations. Organ. Behav. Hum. Decis. Process..

[34] Gibbons F.X., Buunk B.P. (1999). Individual differences in social comparison: Development of a scale of social comparison orientation. J. Personal. Soc. Psychol..

[35] Buunk A.P., Zurriaga R., Peiro J.M. (2010). Social comparison as a predictor of changes in burnout among nurses. Anxiety Stress Coping.

[36] Cumming J., Clark S.E., Ste-Marie D.M., McCullagh P., Hall C. (2005). The functions of observational learning questionnaire (FOLQ). Psychol. Sport. Exerc..

[37] Vealey R.S. (1986). Conceptualization of sport-confidence and competitive orientation: Preliminary investigation and instrument development. J. Sport. Exerc. Psychol..

[38] Martens R., Vealey R.S., Burton D. (1990). Competitive Anxiety in Sport.

[39] Hayes A.F. (2007). Introduction to Mediation, Moderation, and Conditional Process Analysis: A Regression-Based Approach.

[40] Bin W. (2000). A review of the study of attribution retraining. Sport. Sci..

[41] Gordon R.A. (2008). Attributional style and athletic performance level: Strategic optimism and defensive pessimism. Psychol. Sport. Exerc..

[42] Greenlees I., Lane A., Thelwell R., Holder T., Hobson G. (2005). Team-referent attributions among sport performers. Res. Q. Exerc. Sport..

[43] McAuley E., Duncan T.E., Russell D.W. (1992). Measuring causal attributions: The revised causal dimension scale (CDS II). Person. Soc. Psychol. Bull..

[44] Hepler T.J., Chase M.A. (2008). Relationship between decision-making self-efficacy, task self-efficacy, and the performance level of a sport skill. J. Sports Sci..

[45] Moritz S.E., Feltz D.L., Fahrbach K.R., Mack D.E. (2000). The relation of self-efficacy measures to sport performance level: A meta-analytic review. Res. Q. Exerc. Sport..

[46] Smith R.H., Suls J., Wheeler L. (2000). Assimilative and contrastive emotional reactions to upward and downward social comparisons. Handbook of Social Comparison: Theory and Research.

[47] George T.R., Feltz D.L., Chase M.A. (1992). Effects of model similarity on self-efficacy and muscular endurance: A second look. J. Sport. Exerc. Psychol..

